# Two-stage ship detection at long distances based on deep learning and slicing technique

**DOI:** 10.1371/journal.pone.0313145

**Published:** 2024-11-19

**Authors:** Yanfeng Gong, Zihao Chen, Jiawan Tan, Chaozhong Yin, Wen Deng

**Affiliations:** School of Shipping and Naval Architecture, Chongqing Jiaotong University, Chongqing, China; Jiangsu Open University, CHINA

## Abstract

Ship detection over long distances is crucial for the visual perception of intelligent ships. Since traditional image processing-based methods are not robust, deep learning-based image recognition methods can automatically obtain the features of small ships. However, due to the limited pixels of ships over long distances, accurate features of such ships are difficult to obtain. To address this, a two-stage object detection method that combines the advantages of traditional and deep-learning methods is proposed. In the first stage, an object detection model for the sea-sky line (SSL) region is trained to select a potential region of ships. In the second stage, another object detection model for ships is trained using sliced patches containing ships. When testing, the SSL region is first detected using the trained 8th version of You Only Look Once (YOLOv8). Then, the SSL region detected is divided into several overlapping patches using the slicing technique, and another trained YOLOv8 is applied to detect ships. The experimental results showed that our method achieved 85% average precision when the intersection over union is 0.5 (AP_50_), and a detection speed of 75 ms per image with a pixel size of 1080×640. The code is available at https://github.com/gongyanfeng/PaperCode.

## 1. Introduction

With the development of artificial intelligence, the intelligent ship has attracted more and more attention. It can reduce transportation costs and increase navigation security. Visual perception is the eye of intelligent ships. To guarantee the safety of intelligent ships, precise ship detection is essential. The farther away a ship can be detected, the safer the intelligent ship will be. Nearby ships can be detected easily, but when the ship is farther away, the detection results become worse. Especially when the ship is at long distances, it is difficult to obtain accurate features due to limited pixels. In this paper, we focus on detecting ships at long distances at sea.

According to the way images are obtained, ship detection methods are usually categorized into three categories: visual images, radar images, and remote sensing images. Radar is the instrument with the longest distance for ship detection. These are widely deployed aboard ships. However, radar images have limited features, making them challenging to recognize using machine vision. While methods based on remote sensing images are always time-consuming. Visual images have rich features and are the most widely used in intelligent ship technology [1]. Ship detection methods in visual images are usually divided into two types: traditional and deep-learning methods. Ships at long distances in marine environments often appear near the SSL. Thus, the traditional method usually consists of four steps: preprocessing, SSL detection, ROI extraction, and recognition [2]. Deep-learning methods are usually based on convolutional neural networks (CNN). Unlike general object detection in terrestrial environments, ship detection is performed in complex water surface backgrounds that are affected by seabirds, light, wind, water waves, and other factors. This presents significant challenges.

In the early stages, ship detection focused on traditional image-processing methods [3–6]. These methods usually fetch manual pre-defined features to find ships in images. The handcrafted features vary with the distance of the ship, the type of ship, and the weather, making them not robust in applications. Since the images with SSL background have a boundary between the sky and the ocean, and ships at long distances usually appear near SSL, most ship detection methods for these images detect the SSL first and then recognize the surrounding ships [7–9]. In recent years, deep learning has achieved great success in object detection and has attracted increasing attention in ship detection [10–14]. Compared with traditional methods, deep-learning-based methods can automatically obtain features and are more robust. However, there are several difficulties in applying deep-learning-based methods to intelligent ship technology. First, when the ships are at long distances, they appear very small in images and have a similar size to seabirds, clouds, and some water waves. Additionally, they have a similar feature to close-range buoys, making the detection results susceptible to interference from these deceptive objects. Second, the ships at long distances are in low contrast in bad weather, such as fog and rain. Finally, the long-distance ship detection using deep learning lacks sufficient sample data to train the model.

Though some studies have attempted to reduce the impact of seabirds, clouds, and fog [8–9], these methods are not robust enough and have limited effectiveness. Some other works focus on the small ship detection [15–17], most of them are used for remote sensing images, which are from a top-down perspective, unlike the frontal perspective of our long-distance ship images. Besides, to improve the accuracy of small targets, some models have been proposed [18–20]. These methods will be used for comparison with ours in later experiments.

To sum up, the robustness of traditional image-processing methods is poor, and the accuracy of deep-learning methods needs to be improved.

To address ship detection at long distances in marine environments, we proposed a two-stage method that combines the advantages of traditional and deep-learning methods to make full use of the SSL and deep-learning methods. Specifically, we first detected the SSL region in a high-resolution image using a lightweight and accurate object detection model because the SSL is an obvious feature of these images, which are captured over long distances. Second, YOLOv8 combined with the slicing technique was utilized to detect ships in the resulting SSL region, where ship features did not need to be compressed in the input images.

The main contributions of this study are as follows.

1. We constructed a long-distance ship dataset, which is applicable for the visual perception of intelligent ships (available at https://drive.google.com/drive/folders/1WKjZYdarcy4PYKcjg0idqQDWStrPLvUv?usp=sharing).
2. A novel ship detection method at long distances was proposed. The proposed method divides the detection of ship targets into two steps: SSL detection and ship detection, greatly improving the ship detection accuracy while ensuring detection speed.
3. Ship detection combined with slicing technique was proposed, greatly improving the small ship detection accuracy.
4. Our method is suitable for high-resolution image capture devices, which can obtain high-quality images of ships at long distances, and is more conducive to visual perception of intelligent ships.

In this paper, Section 2 introduces the related work that is relevant to our method. Section 3 presents the proposed method. Section 4 details the experiments and their corresponding results. Section 5 outlines the conclusions.

## 2. Related works

### 2.1. Ship detection

Before deep learning became popular, ship detection at long distances focused on traditional image-processing methods. Zhang et al. [21] detected the SSL using a discrete cosine transform first, then completed background modeling and background subtraction for ship detection. Liang et al. [9] and Lin et al. [6] utilized gradient features to detect SSL for ship detection to prevent the influence of local noise; however, they had to choose the appropriate handcrafted features. Occasionally, impacted by different weather conditions, ship images at sea may be in low contrast, and the accuracy of SSL and ship detection is poor in this condition. Shan et al. [7] used SSL and saliency detection to alleviate this effect of the weather. Except for the weather, the detection results are easily affected by sea clutter. A frequency-domain method was adopted for ROI extraction when the ships were in such scenarios [5]. This method alleviates this influence. This is not applicable when the sea clutter is strong. Li et al. [4] used prior knowledge, such as aspect ratio, contrast ratio, ship size, and grayscale, to identify ships around the SSL and improve detection accuracy. However, these features are handcrafted and are not robust enough. Therefore, adopting traditional methods for complex scenes is challenging and requires selecting appropriate handcrafted features, which exhibit poor robustness.

With the development of neural networks, deep-learning-based methods have attracted increasing attention in ship detection. At present, there are two kinds of deep learning-based object detection methods: (a) two-stage detectors, such as SPP-Net [22], fast R-CNN [23], and faster R-CNN [24]; (b) one-stage detectors, such as You Only Look Once (YOLO) series [25–31], SSD [32], RetinaNet [33], and CenterNet [34]. Marie et al. [35] described a fast R-CNN-based maritime object-detection method that achieved satisfactory results. This method divided the detection of ship targets into three steps, but they did not consider SSL. Qi et al. [36] proposed an improved faster R-CNN algorithm for ship detection that significantly shortened the detection time while improving the detection accuracy. However, these methods are time-consuming. To improve the detection speed and accuracy simultaneously, many researchers have proposed variants based on object detection models. Shan et al. [37] proposed a novel method called SiamFPN for ship detection at sea. Wang et al. [38] presented an improved YOLOv3 algorithm that realizes an end-to-end ship target detection system, making the application of deep-learning methods in ship detection feasible. Lee et al. [39] proposed an improved one-stage model that realizes floating object detection, including ships at sea, at 30 frames per second. This method did not take into account the ship’s detection at long distances and the influence of weather. To reduce the influence of the weather, Liu et al. [40] proposed an enhanced CNN, improving ship detection. Wang et al. [11] constructed a real-time ship target detection method based on YOLOv4, improving the detection speed and accuracy. They also did not consider ship detection at long distances. Zhang et al. [41] and Qin et al. [42] presented improved methods based on YOLOv3. Their accuracy was satisfactory for sea-surface ships of general sizes. Li et al. [43] proposed a ship detection and recognition method based on a multilevel hybrid network combining traditional image processing and deep-learning methods to improve detection speed. Xu et al. [44] proposed LMO-YOLO to address the high false detection rate of ships in low-resolution images. Liu and Zhu [45] proposed a residual YOLOX-based ship object detection model, that is applicable to ship image detection in ports. This method focuses on the ship detection of optical satellite images, where images do not have SSL. Zhou et al. [13] present an improved YOLOv5 model for ship detection, greatly increasing the detection speed. Wang et al. [31] proposed YOLOv10 model based on the previous version of YOLO. It further improved the performance of YOLO. These deep-learning-based methods have achieved good results for ship detection. However, they are not applicable to ship targets over long distances at sea, which is a small-target detection problem.

Long-distance ship target detection is a subcategory of generic ship target detection and has attracted increasing attention. Wei et al. [46] presented a small-target detection method based on hierarchical and multi-scale convolutional neural networks aiming at detecting maritime targets in complex scenarios. The detection speed needs to be improved. Chen et al. [47] proposed a novel hybrid deep-learning method that combined a modified generative adversarial network (GAN) and a convolutional neural network (CNN)-based detection approach for small-ship detection. This method can alleviate the overfitting caused by insufficient sample data. However, it is not suitable for ship detection at long distances. Nina et al. [48] compared YOLO and YOLT in detecting small ship objects. These results indicate that the accuracy of both YOLO and YOLT for small ship objects must be improved. Yu et al. [49] present a modified YOLOv3 model for small-scale ship target detection, improving both recall rate and accuracy rate. Hu et al. [50] proposed a novel small-ship detection method based on YOLOv4 to solve the problem in which global and local relationships in the input image are rarely considered. Escorcia-Gutierrez et al. [51] presented an efficient optimal mask-CNN technique for small-ship detection using autonomous shipping technologies and obtained satisfactory results. Similarly, these methods only considered small target detection caused by the small size of the ship itself and did not consider the scenario when the ship is far away. Recently, Wang et al. [52] and Qu et al. [53] combined the YOLOX algorithm with a convolutional block attention module (CBAM) to improve the accuracy of small ship targets. Experiments showed that CBAM can fetch better features for small ships. Sun et al. [16] proposed a novel model for small object detection. However, this method focused on the small ship detection from the bird’s eye view, which is not suitable for the visual perception scenario of intelligent ships. Guo et al. [17] constructed a multi-scale key point-based detector to improve tiny object detection. These methods can obtain more fine-grained features. However, to detect ships at longer distances, high-resolution cameras are usually used, acquiring images with a pixel size much larger than 640×640. This means that images fed into the model must be compressed, losing many valuable features.

### 2.2. YOLOv8 model

Among the deep-learning methods, the YOLO series have attracted more and more attention. These methods are one-stage CNN-based detectors. Compared to two-stage detectors, they have a better trade-off between accuracy and detection speed. As of now, the latest version of YOLO is YOLOv10 [31]. But when we started this work, the most popular YOLO was YOLOv8 [30]. YOLO [25] is the first version of the YOLO series. It directly maps each feature pixel of the grid cell to bounding boxes and class probabilities that consider the global information of the input image, resulting in a faster detection speed. In addition, YOLO sets a precedent for anchor-free object detection. As the superiority of the anchor boxes strategy in two-stage detectors, Redmon and Farhadi proposed YOLOv2 [26], which adopted the anchor boxes strategy again and introduced Multi-Scale Training, improving the accuracy of YOLO. To further improve its performance, Redmon and Farhadi [27] proposed YOLOv3 by introducing ResNet, FPN, Adam optimizer, and independent logistic classifiers. Based on YOLOv3, YOLOv4 [28] adopts mosaic and cut mix data augmentation, CSPNet [54], CIOU [55], and label smoothing. In the later improved versions, YOLOv5 [29] and YOLOv8 [30] were used most frequently. YOLOv5 introduces an adaptive anchor box and neighborhood positive and negative sample allocation strategies based on YOLOv4, accelerating convergence. Compared with YOLOv5, YOLOv8 has a decoupled head and is anchor-free. Additionally, it replaces CSPNet with a C2f structure and adopts a distribution function loss with a richer gradient flow.

YOLOv8 consists of three parts: backbone, neck, and head. The backbone was used to extract features, and the C2f structure and SPPF were adopted. The multiscale features were fused using the neck. Decoupled structures are utilized by the head for classification and bounding-box regression.

**Fig 1:**
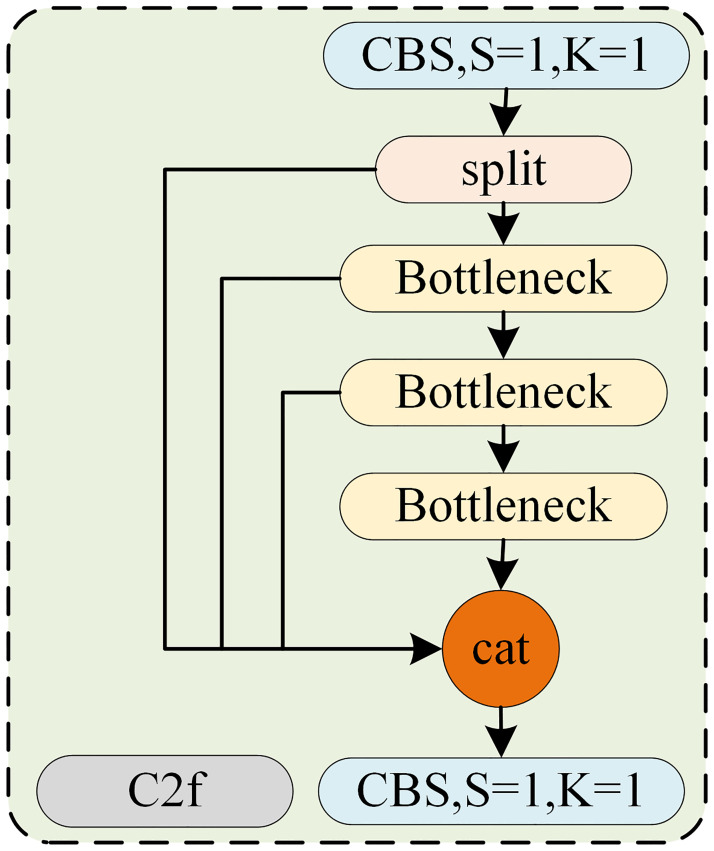
Structure of C2f used in YOLOv8.

The biggest highlight of YOLOv8 is the use of the C2f structure, which is improved based on the idea of CSPNet. The C2f structure is shown in Fig 1, the gradients on the right-hand side were separately integrated. In contrast, the feature map on the left side was integrated separately. Neither side contained duplicate gradient information. Thus, it preserves the advantages of the feature reuse characteristics on the right side and simultaneously prevents excessive duplicate gradient information by truncating the gradient flow. The C2f structure has the following three advantages: 1) it can enhance the study ability of the model by simultaneously making the model lightweight and improving its accuracy, 2) it can reduce the computation bottleneck, and 3) it can reduce the memory costs.

## 3. Proposed method

**Fig 2:**
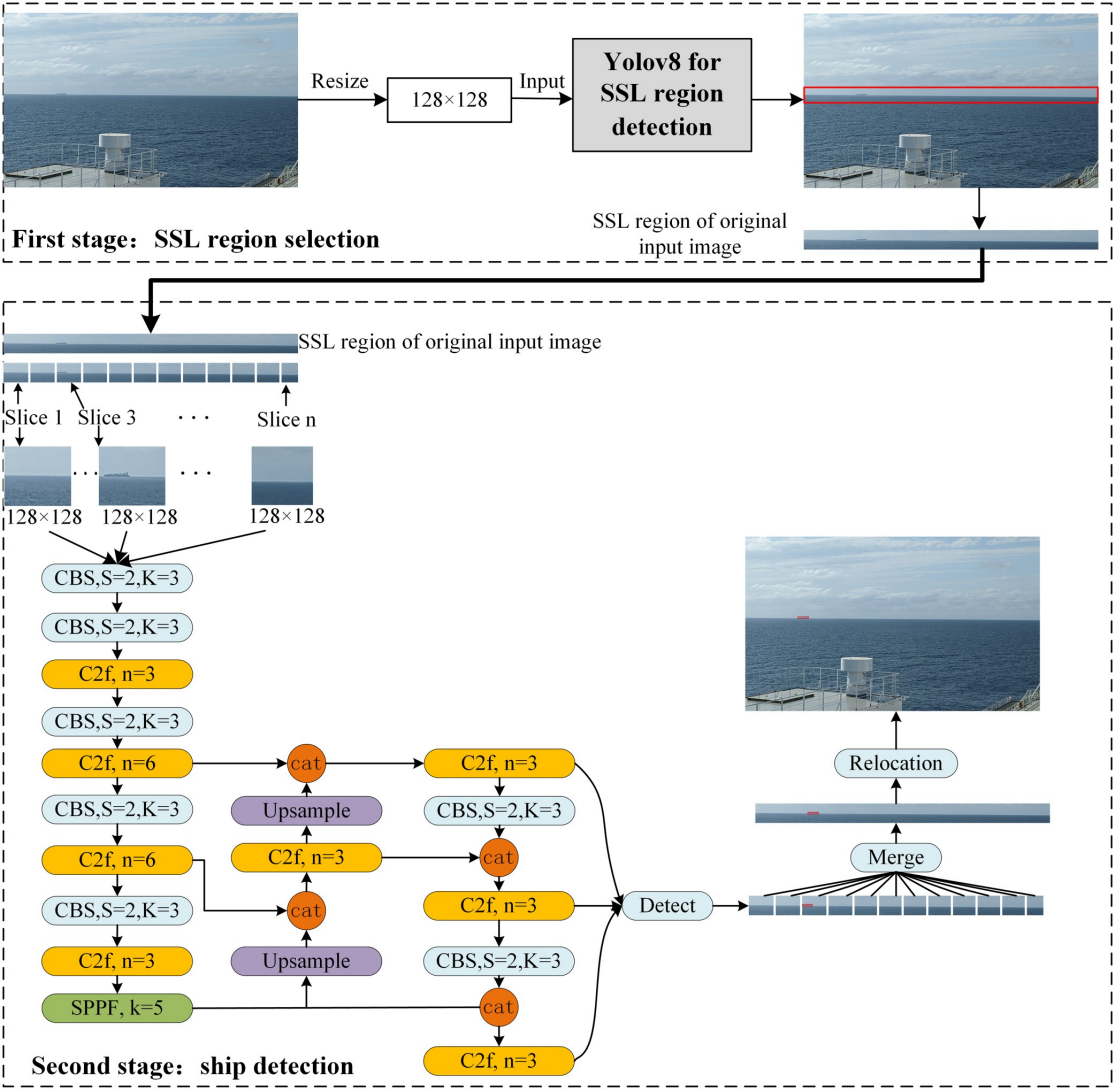
Overview of the proposed long-distance ship detection framework.

In this study, we proposed a two-stage YOLOv8 for long-distance ship detection at sea. The architecture of the proposed two-stage YOLOv8 is shown in Fig 2. It consists of two stages: the selection of the SSL region and ship detection. Additionally, we constructed a ship dataset suitable for the visual perception of intelligent ships.

### 3.1. Ship dataset

**Fig 3:**
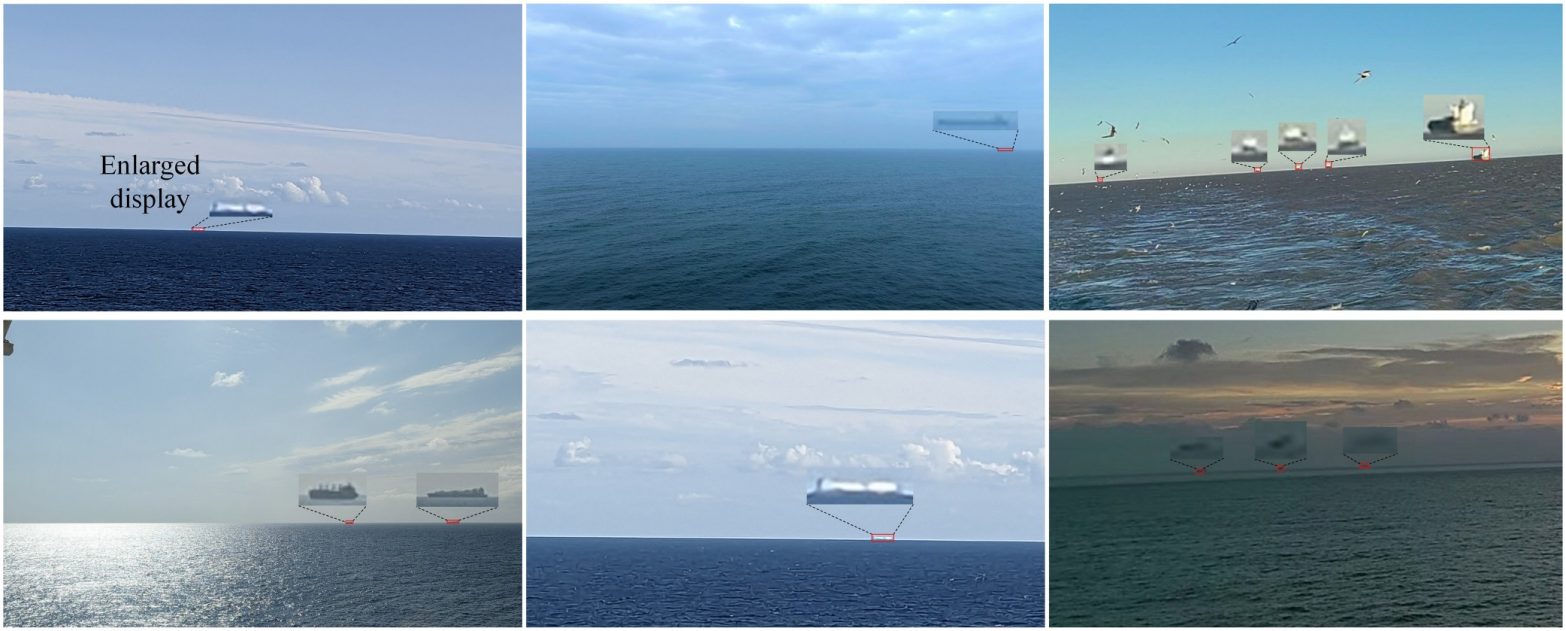
Presentation of ship samples in our dataset.

**Fig 4:**
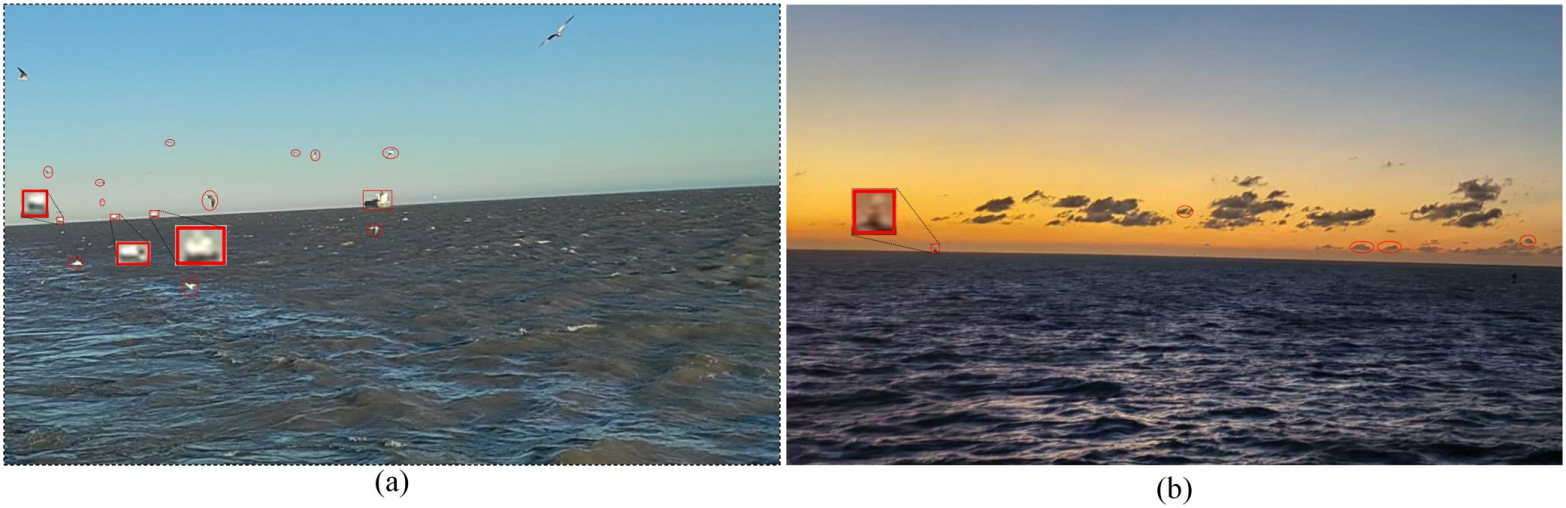
Examples of difficult-to-identify samples. **(a)** True ship targets are circled by rectangles, and false ship targets circled by ellipses are seabirds; **(b)** true ship targets are circled by rectangles, and false ship targets circled by ellipses are clouds.

There are some public datasets of visual images for ship detection [56–58], but they are not from the perspective of long-distance, where SSL is not present in all images. And there is no unified public dataset for long-distance ship target detection. We collected a ship dataset containing 1871 ship images. When capturing pictures, the target ships were far away from the optical camera. This is applicable in the context of intelligent ship technology, where two ships are at a considerable distance from each other. As a result, the ship in each captured image is positioned close to the SSL. As shown in Fig 3, the ship appears very small because of its long distance from the camera, and it is near the SSL. Over long distances, seabirds and clouds can easily be mistaken for ships. The parts circled in the ellipses are seabirds, as shown in Fig 4a. In Fig 4b, the areas circled in the ellipses are clouds. All have features similar to those of ships over long distances. Additionally, waves formed on the sea surface by strong winds are highly similar to distant ships. Samples with these characteristics were collected to demonstrate the superiority of our method. The dataset was divided into two parts, the training set and the validation set, with a ratio of 9:1. At the same time, the validation set was also used as the test set. The details of the dataset are listed in Table 1.

**Table 1:** Presentation of our ship dataset.

Item	Number
Training set	1683
Validation set (Test set)	188
Total	1871
Ships	4348

**Fig 5:**
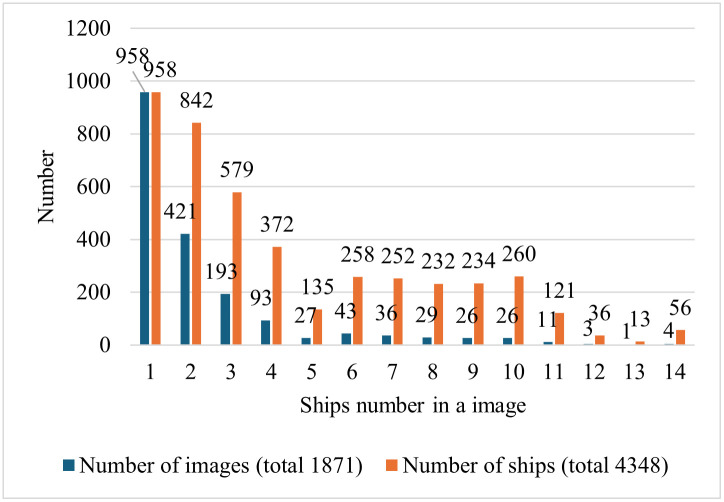
Illustration of ship targets in each image sample. For example, for the second cylinder cluster, there are 421 images, each of which contains two ship targets, a total containing 842 ship targets.

**Fig 6:**
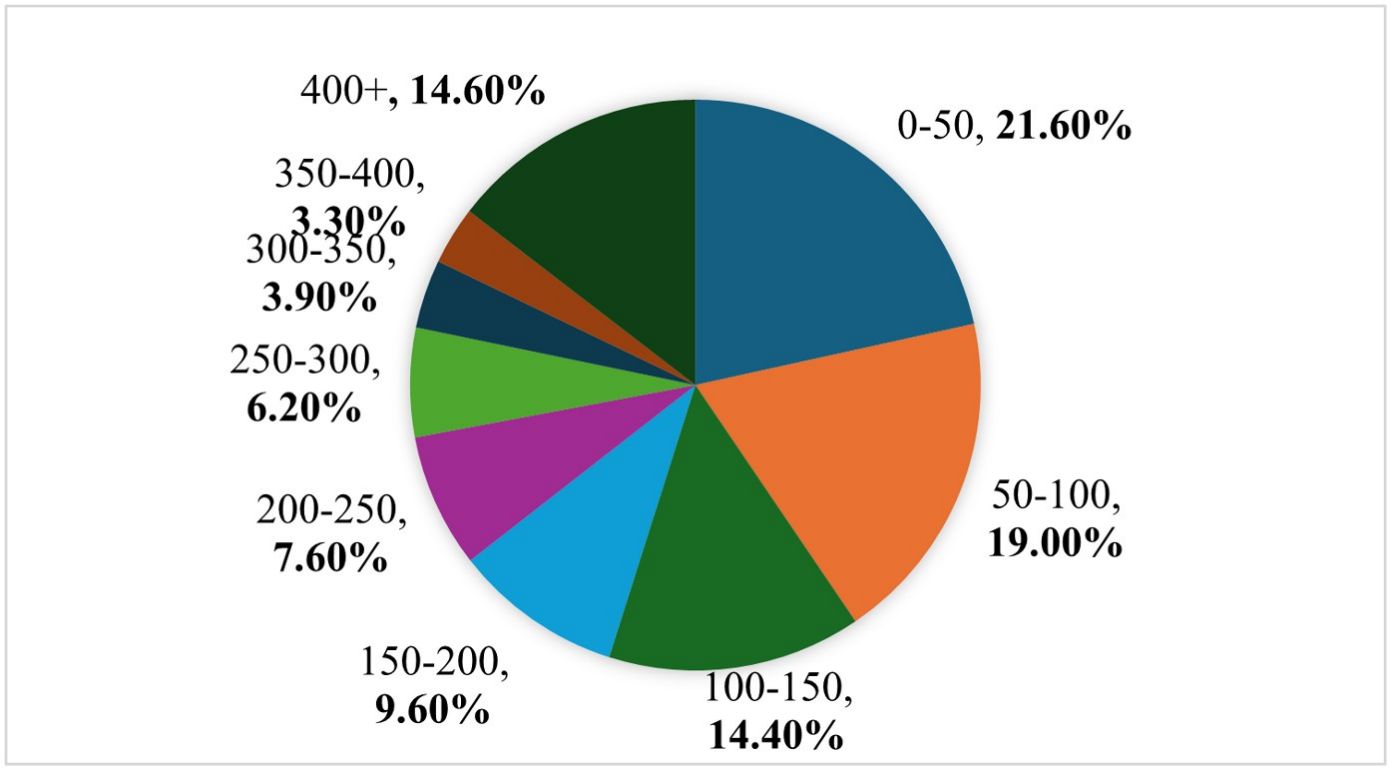
Statistics of bounding box areas in pixels.

**Fig 7:**
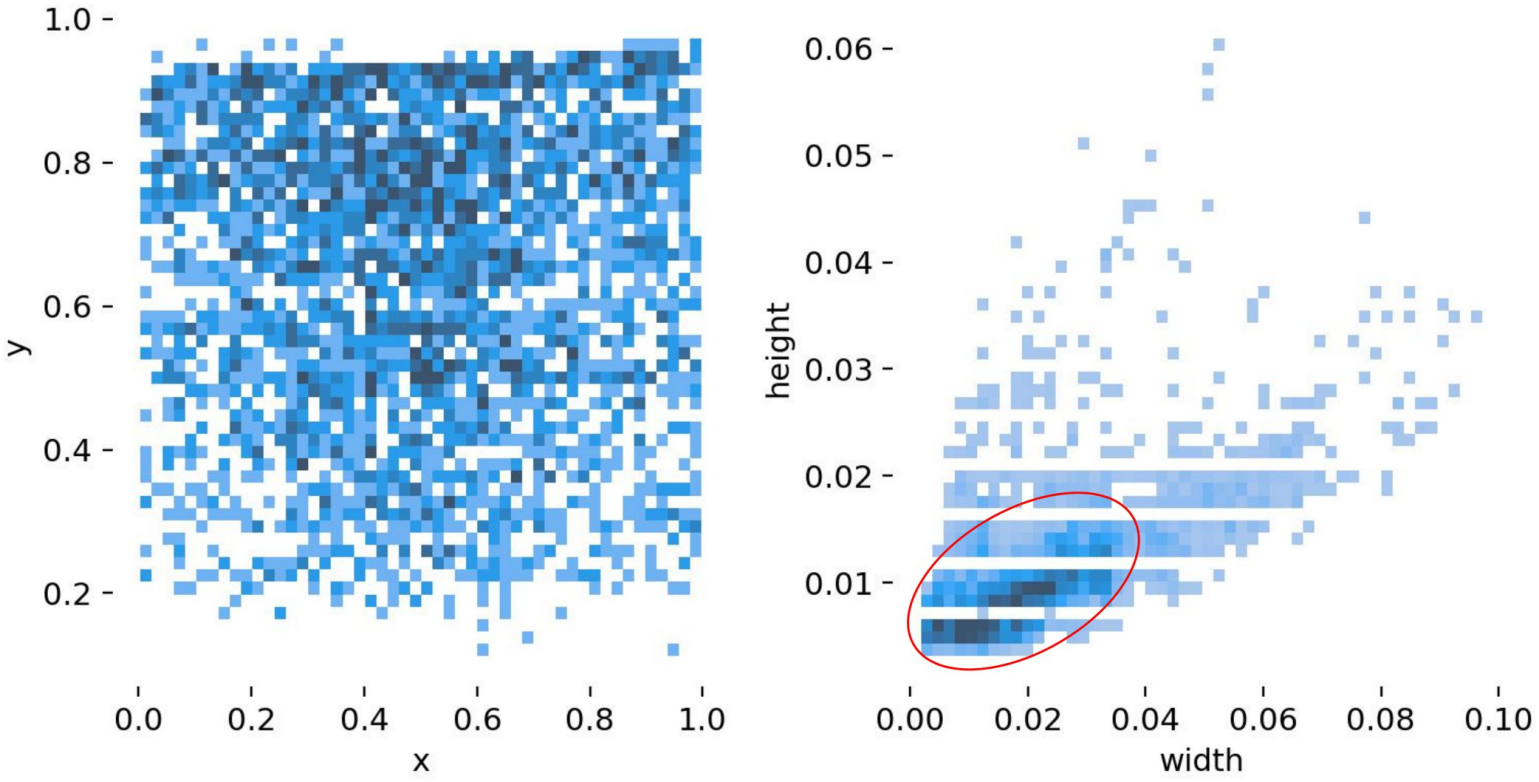
Coordinate and size statistics of ship bounding box.

Fig 5 illustrates the distribution of ships in the images. There were 4348 ship instances in 1871 image samples. The number of ships in each image ranged from 1 to 14. Most images contain only one ship instance. The number of image samples was 958, as shown in the first cylindrical cluster in Fig 5. For the last cylinder cluster in Fig 5, there are four images, each of which contains 14 ship instances, for a total of 56 ship instances. Fig 6 shows the statistics of the ship bounding boxes in the dataset, including the coordinates and size distribution. According to the definition of small targets in the MS COCO dataset, targets smaller than 32 × 32 pixels (1024 pixels) are called small targets. In our dataset, 21.6% of the objects were smaller than 150, and 85.4% were smaller than 400. Most instances have a width less than 0.03 of the image width and a height less than 0.01 of the image height, as shown in Fig 7. All instances were evenly distributed throughout the images. The average size of the instances in our dataset was 229 pixels, and the smallest size was only four pixels. Thus, it is more challenging than a typical small-target detection problem.

### 3.2. SSL region selection model with YOLOv8

Ships at long distances appear small in the image, usually just taking up several pixels of the whole image, and always lying near the SSL. In contrast, the SSL region is conspicuous, spans the image from left to right, and makes it easily detectable. Most object detection models can detect SSL from images accurately, whereas long-distance ships are difficult to detect. Thus, if the SSL region is selected correctly, a ship can be detected using a suitable method.

Because of the excellent characteristics of YOLOv8, which has good speed and accuracy, we adopted it to detect the SSL region in our first stage.

The loss in the SSL region selection model consists of two parts: rectangular box loss and classification loss, where rectangular box loss includes *CIOU* loss and *DFL* loss. The total loss is defined as

$$Loss=\left(a*CIOU+b*DFL\right)+c*loss_{cls}$$ (1)

where *CIOU* and *DFL* are the rectangular box loss and *loss*_*cls*_ is the classification loss. The *a*, *b*, and *c* are the weight coefficients of each loss. In general, *a* is 7.5, *b* is 1.5, and *c* is 0.5.

BCELoss with sigmoid was adopted to compute *loss*_*cls*_, which is denoted as

$$loss_{cls}=\frac{1}{N}\sum_{i}^{N}\left[-y_{i}\;\text{log}\left(p_{i}\right)-\left(1-y_{i}\right)\text{log}\left(1-p_{i}\right)\right]$$ (2)

where *p*_*i*_ is the probability of the *i*-th ship predicted, and *y*_*i*_ is the label of the *i*-th ship. *N* represents the total number of ship samples.

**Fig 8:**
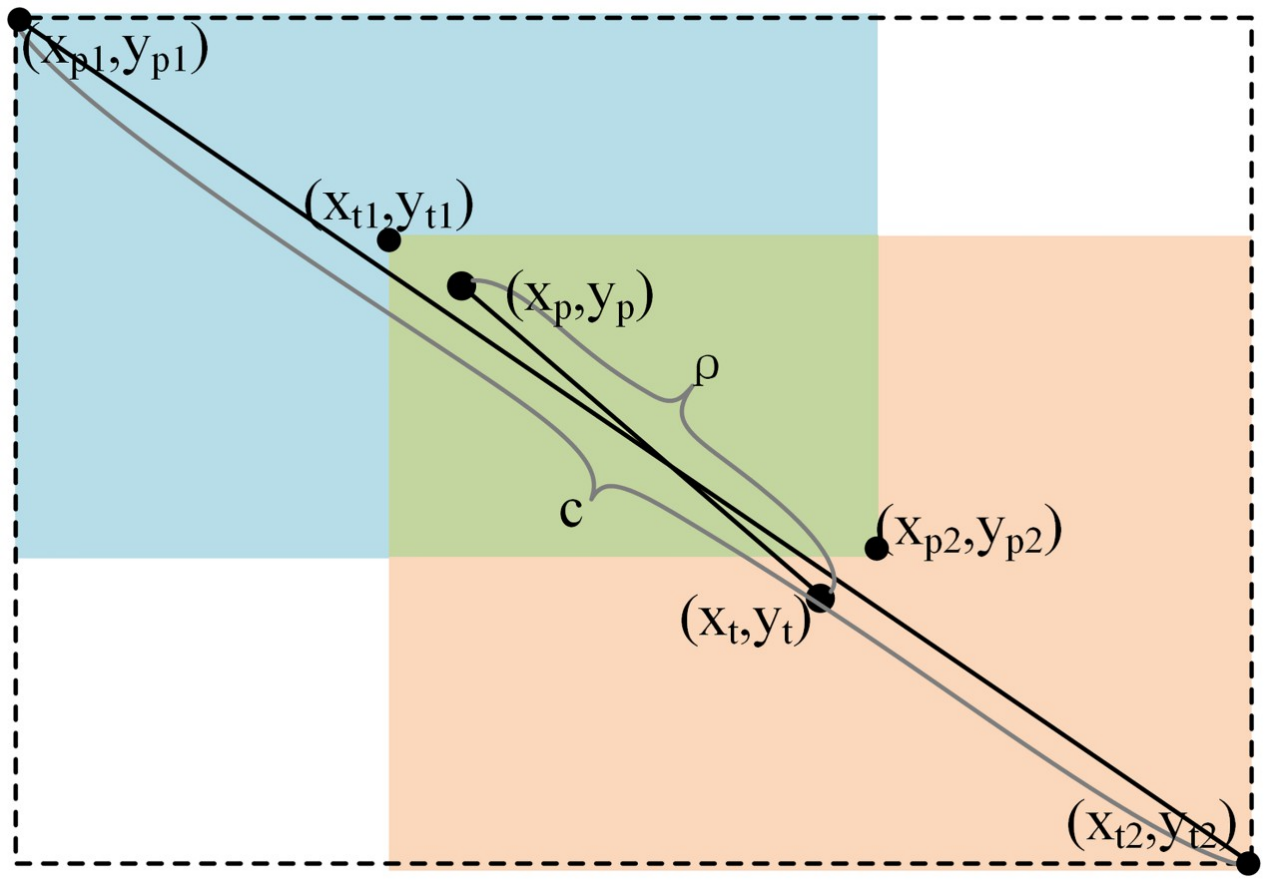
Description of CIOU.

The *CIOU* loss and *DFL* loss are given by

$$CIOU=IOU-\frac{\rho^{2}}{c^{2}}-\alpha v$$ (3)

$$DFL\left(S_{i},S_{i+1}\right)=-\left(\left(y_{i+1}-y\right)\;\text{log}\left(S_{i}\right)+\left(y-y_{i}\right)\text{log}\left(S_{i+1}\right)\right)$$ (4)

where *S*_*i*_ and *S*_*i+*1_ denote the prediction probability of the left integer point and the right integer point of the corresponding anchor point respectively. As shown in Fig 8, *IOU* is the intersection ratio of two bounding boxes, *ρ* is the distance between two bounding boxes’ center points, *c* is the distance between the left-up point and right-bottom point of the bounding rectangle of two bounding boxes, *v* is the ratio of width and height of two bounding boxes, *α* is the coefficient of *v*. The four parameters are defined as

$$\left\{\begin{array}{c} IOU=\frac{S_{1}}{S_{2}}\\ \rho^{2}={\left(x_{p}-x_{t}\right)}^{2}+{\left(y_{p}-y_{t}\right)}^{2}\\ c^{2}={\left(\text{max}\left(x_{p2},x_{t2}\right)-\text{min}\left(x_{p1},x_{t1}\right)\right)}^{2}+{\left(\text{max}\left(y_{p2},y_{t2}\right)-\text{min}\left(y_{p1},y_{t1}\right)\right)}^{2}\\ v=\frac{4}{\pi^{2}}{\left(arctan\frac{x_{t2}-x_{t1}}{y_{t2}-y_{t1}}-arctan\frac{x_{p2}-x_{p1}}{y_{p2}-y_{p1}}\right)}^{2}\\ \alpha =\frac{v}{1-IOU+v} \end{array}\right.$$ (5)

where (*x*_*p*_,*y*_*p*_) and (*x*_*t*_,*y*_*t*_) are the center points of the predicted and true bounding boxes, respectively. (*x*_*p*1_,*y*_*p*1_) and (*x*_*p*2_,*y*_*p*2_) are the bottom-left and right points of the predicted bounding box, respectively. (*x*_*t*1_,*y*_*t*1_) and (*x*_*t*2_,*y*_*t*2_) are the upper left and bottom right points of the true bounding box, respectively. *S*_1_ and *S*_2_ are the intersection and union areas of the two bounding boxes, respectively.

The SSL generally spans the entire width of the image, with the upper part being the sky and the lower part being the ocean. Because of these distinctive features, we usually resized the input image to 128 × 128 pixels. It achieved almost the same detection accuracy as when using an input size of 640 × 640 pixels, while significantly improving the detection speed. This setting was verified in subsequent experiments.

An apparent advantage of detecting the SSL region is that it eliminates the negative influence of clouds and seabirds in the sky, as well as the influence of waves on the sea when detecting ships near the SSL.

### 3.3. Ship detection with YOLOv8 and slicing technique

After detecting the SSL region, a suitable method must be selected to detect ships in the region. The width of the SSL region is significantly greater than its height. Existing object detection models usually require a specified size for the input images and are not suitable for the SSL region. In the YOLO series, an image is resized to the same height and width as the input to the model. For YOLOv2, the input size is 448 ×448 pixels. For YOLOv5 and YOLOv8, the input size was 640 × 640. For the faster R-CNN, although images are required to be resized to a specified ratio to input the model, the SSL region with a large aspect ratio is not suitable for training a model, which could cause serious distortion of the ship target. In this study, we adopted the slicing technique. Using this technique, an image is sliced into several patches. All patches of an image are input into the ship object detection models, and the detection results are combined into one in the original image.

**Fig 9:**
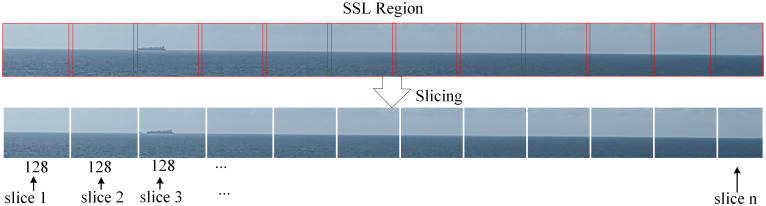
Description of slicing technique.

**Fig 10:**
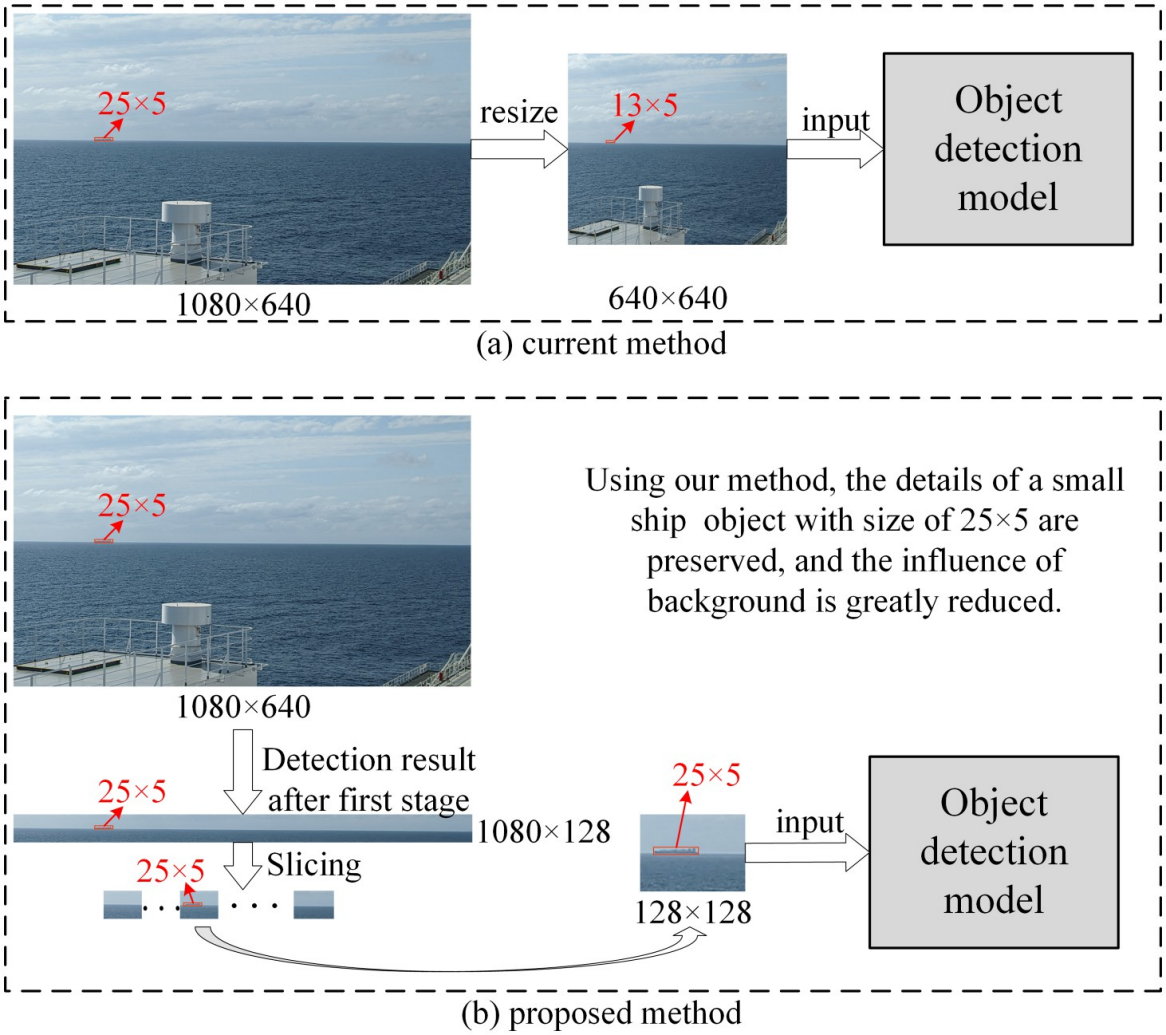
Comparison of current methods and our proposed method used in object detection. (a) Current methods and (b) proposed method.

The slicing technique is illustrated in Fig 9. The width of the SSL region is the same as that of the original input image, whereas its height is usually between a few and dozens of pixels, according to the size of the ships in it. Thus, we cropped the SSL region into patches whose heights were the same as those of the SSL region, and whose widths were fixed at 128 pixels. These patches are then input into an object detection model. After obtaining all patch results of an SSL region, we combined these results into one, denoting the final detection result of an input image. There is an obvious advantage to using the slicing technique. Ships in the SSL region comprise several pixels in the original high-resolution image, which is typically much larger than 640 × 640 pixels. After an image was resized to 640 × 640 pixels before being input to the model, the ships in it became smaller, making it more difficult to detect. However, using the slicing technique, the original small ship objects do not need to be resized to a smaller size. Each patch had the same resolution as the SSL region. When these patches were input into an object detection model, they were resized to 128 × 128 pixels using interpolation. This made the original small ship object easier to detect. As shown in Fig 10a, a ship over long distances occupies only 25 × 5 pixels in a high-resolution image of size 1080 × 640. If the current methods are used, they are resized to 13 × 5 pixels before inputting the object detection model. While the size 25×5 is preserved before inputting the object detection model in our method, as shown in Fig 10b. Not only are the features of small ships retained but the influence of background is greatly reduced. In this study, we chose YOLOv8 as the object detection model because of its excellent small-object detection accuracy and good detection speed.

After all the patches of an SSL region were detected, their detection results were combined into one displayed in the SSL region. Subsequently, based on the location of the SSL region in the original image, the ships detected in the SSL region continue to be located in the original image, as shown in Fig 2.

## 4. Experiments

In this section, we present extensive experiments conducted to demonstrate the superiority of the proposed method. First, the experimental environment and setup are introduced. Subsequently, the metrics are presented. Finally, two types of experiments are presented: methods with and without our two-stage strategy, and comparisons with state-of-the-art models.

### 4.1. Experimental environment and setup

All experiments were conducted using a PyTorch framework on a computer with a 64-bit Ubuntu-16.04 operating system. The GPU used was an NVIDIA GeForce RTX 3090ti with an Intel Core i7-10700 CPU. The Python version was 3.9.18, the Torch version was 1.8.0, and the CUDA version 11.1. The specific configurations are listed in Table 2. This is because the proposed method comprises two models with different settings. In the first stage, we adopted YOLOv8 with a batch size of 8. The input size is 128 × 128 pixels. In the second stage, we adopted YOLOv8 as the ship detection model. The input images were patches with a batch size of 8 and an input size of 128 × 128. Other hyperparameters, including the learning rates and weight decay, in these two stages were set by default, which were the same as the original settings in YOLOv8 [30].

**Table 2:** Configuration of experimental environments.

Parameter	Configuration
Operating system	Ubuntu16.04
Python	3.9.18
GPU	NVIDIA RTX3090TI
CPU	Intel core i7-10700
CUDA	Version 11.1
Torch	Version 1.8.0
Torchvision	Version 0.9.0

### 4.2. Evaluation criterion

Several state-of-the-art methods have been used to conduct comparative experiments and demonstrate the superiority of the proposed method. Therefore, we have selected an evaluation criterion. Accuracy and speed are the two main metrics used in object detection methods.

Accuracy is measured using the average precision (*AP*). *AP* is defined as the area under the precision-recall curve. The precision and recall are expressed as

$$Precision=\frac{TP}{TP+FP}$$ (6)

$$Recall=\frac{TP}{TP+FN}$$ (7)

where TP is the number of bounding boxes whose predicted labels are the same as the ground truth, FP is the number of bounding boxes whose predicted labels differ from the ground truth, and FN is the number of bounding boxes where the ground truth is not predicted.

For multiclass object detection, the mean value of all the *AP* is calculated and is called the mean average precision (mAP). In this study, mAP specifically refers to the *AP* of a single class. The formulas for the *AP* are as follows:

$$AP=\int_{0}^{1}P\left(R\right)dR$$ (8)

where *P*(*R*) is the precision predicted at the recall *R*. The *AP* values ranged from 0 to 1. Different *IOU* has different *AP*, where *IOU* ranges from 0.5 to 0.95 with step 0.05. As most instances in our dataset are extremely small, a higher *IOU* can lead to poor detection results. We chose *IOU* 0.5 when computing the *AP*, which is written as AP_50_.

In addition to measuring the *AP*, we measured the speed of the proposed method. In this study, we computed the inference time for images in one batch size to obtain the time required to handle one image.

### 4.3. Experiments with and without our two-stage strategy

In this experiment, we evaluated the performance of three typical networks: Faster R-CNN, YOLOv5, and YOLOv8. We applied it to three networks to demonstrate the effectiveness of our two-stage strategy. This indicated that YOLOv8 was first used to detect the SSL region. Subsequently, Faster R-CNN, YOLOv5, and YOLOv8 with the slicing technique were used to detect ships in the SSL region.

**Fig 11:**
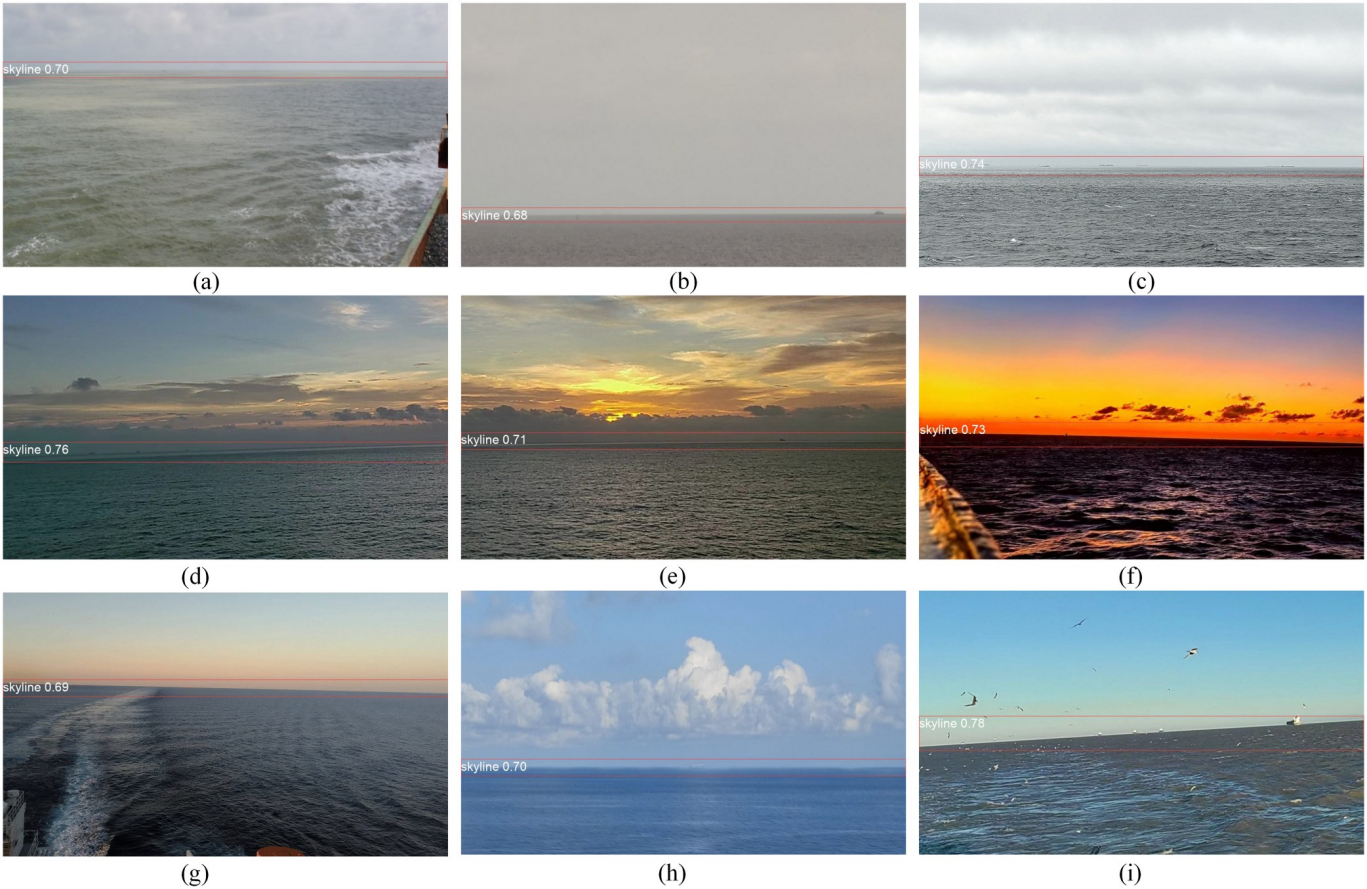
Visualization of the SSL detection results under various conditions. **(a)** overcast; **(b)** overcast and foggy; **(c)** overcast and existing waves; **(d)-(e)** nightfall and cloudy; **(f)-(g)** morning and existing morning glow; **(h)** cloudy and existing waves; **(i)** existing seabirds.

In the first stage, the size of the input image is set to 128 × 128 pixels. As shown in Table 3, when the input size of images is set to 640 × 640 pixels, the AP_50_ and AP_50:95_ for SSL using our method are 0.99 and 0.66, respectively. Although the input size is set to 128 × 128 pixels, the AP_50_ and AP_50:95_ become 0.967 and 0.658, which are slightly lower than that of 640 × 640. However, the detection speed has been doubled, from 1.6 ms to 0.8 ms. Although the ship becomes almost invisible in the resized images of 128 × 128 pixels, the SSL region, which always spans the entire image and serves as the boundary between the sky and sea, had little impact on its features. This led to a significant improvement in detection speed compared to an input size of 640 × 640. Fig 11 shows the visualization of the SSL detection results under various conditions. When the weather is overcast, the SSL becomes a little blurry but can be correctly detected using YOLOv8, as shown in Fig 11a–11c. When the light is dim and there are clouds near the SSL, the features of SSL become less obvious, which increases the difficulty of SSL detection. Under this condition, YOLOv8 can also detect the SSL correctly, as shown in Fig 11d and 11e. If the glow appears when the light above the sea is dim, the features of the SSL will be slightly more obvious, as shown in Fig 11f and 11g. In addition to clouds, waves and seabirds can affect the ship detection. Since YOLOv8 could detect SSL correctly, as shown in Fig 11i, seabirds outside the SSL region can be filtered out, thereby improving the accuracy of ship detection.

**Table 3:** Comparison for SSL detection of two different input size.

Metrics	640 × 640 (Input size)	128 × 128 (Input size)
Precision	0.943	0.955
Recall	0.966	0.909
AP_50:95_	0.66	0.658
AP_50_	0.99	0.967
Speed(ms)	1.6	0.8

In the second stage, we detected the ship in the SSL region using the Faster R-CNN, YOLOv5, and YOLOv8 with the slicing technique, to demonstrate the effectiveness of our two-stage strategy. During this stage, the input size of the image patches is set to 128 × 128 pixels. The results are summarized in Table 4. The Faster R-CNN with our two-stage strategy achieved better results than the original model, with a 0.041 improvement in AP_50:95_ and a 0.122 improvement in AP_50_. For the YOLO series models, YOLOv5 combined with our proposed strategy significantly outperformed the baseline YOLOv5, with increases of 0.31 and 0.411 in terms of AP_50:95_ and AP_50_, respectively. When combined with YOLOv8, AP_50:95_ and AP_50_ performed better, reaching 48% and 85% accuracy, respectively. The ship features were retained in the original high-resolution images by inputting image patches into the detection model instead of using entire images. In the original models, the ship features were also compressed when the high-resolution images were resized to 640 × 640 pixels. Thus, detection accuracy was significantly improved using our two-stage strategy. The detection time changes from one image to multiple patches because a high-resolution SSL region is divided into several patches, increasing the total detection time. As shown in Table 4, the detection time was approximately six times the original or even more when adopting our two-stage strategy. For the two-stage YOLOv8, the detection speed is 75 ms per image, which has little impact on the visual perception of intelligent ships where ship detection is carried out at a certain interval. Additionally, a terminal carrying a high-performance graphics card can perform real-time detection under these conditions. These experimental results demonstrate the effectiveness of the proposed two-stage strategy.

**Table 4:** Experimental results with and without the two-stage strategy.

Model	Input size	AP_50:95_	AP_50_	Speed(ms)
YOLOv5	640	7.4%	32.8%	27.4
Two-stage YOLOv5	128+128	38.4%	73.9%	150
Faster R-CNN	640	20.7%	39.7%	20.6
Two-stage Faster R-CNN	128+128	24.8%	51.9%	145
YOLOv8	640	41.5%	73.9%	2.1
Two-stage YOLOv8	128+128	**48%**	**85%**	75

### 4.4. Comparison with the state-of-the-art

We compared it with several state-of-the-art object detection models, such as TPH-YOLOv5, YOLO-Fastestv2, YOLOv8, and YOLOv10, to further prove the superiority of the proposed model. The results are summarized in Table 5. Because YOLOv8 makes some improvements based on YOLOv5, making the network lightweight and accurate, it exceeds 0.411 in AP_50_ compared with YOLOv5. In addition, YOLOv8 clearly outperforms Faster R-CNN, CenterNet, and SSD, except for TPH-YOLOv5, YOLO-Fastestv2, and YOLOv10. However, TPH-YOLOv5 and YOLO-Fastestv2 had longer inference times than YOLOv8. Due to the high real-time performance of YOLOv8, with an inference time of just 2.1 ms per image, we selected it as the SSL detector in the first and second stages. Our proposed method is tested using two different settings: two-stage YOLOv8-a and two-stage YOLOv8-b, with input sizes of 640+128 and 128+128, respectively.

**Table 5:** The detection results of the proposed method and other state-of-the-art models.

Model	Input size	AP_50_	Speed(ms)
Faster R-CNN [24]	640	39.7%	20.6
YOLOv5 [29]	640	32.8%	27.4
SSD [32]	640	63.2%	7
CenterNet [34]	640	52.1%	20.7
TPH-YOLOv5 [18]	640	77.2%	47.9
YOLO-Fastestv2 [19]	640	81%	276
YOLOv8 [30]	640	73.9%	2.1
YOLOv10 [31]	640	75%	1.9
Two-stage YOLOv8-a	640+128	**87%**	76
Two-stage YOLOv8-b	128+128	**85%**	75

**Fig 12:**
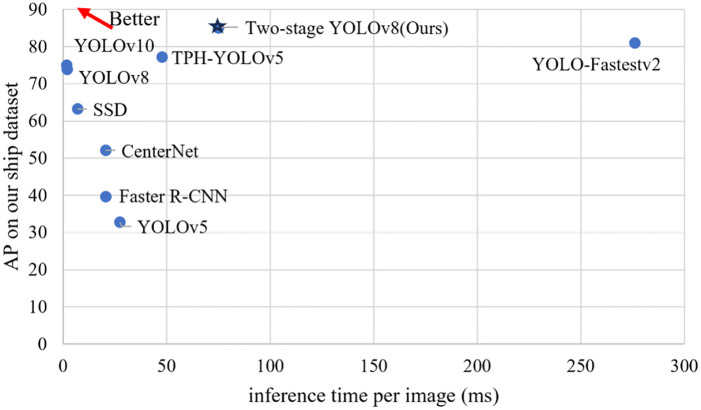
Comparisons of comprehensive performance on our long-distance ship dataset.

In terms of AP_50_, two-stage YOLOv8-a and two-stage YOLOv8-b achieved 87% and 85% accuracy respectively, exceeding that of YOLOv8 by 0.111 and 0.131 respectively. In addition, our method is more accurate than other state-of-the-art models. Specifically, in terms of AP_50_, the proposed two-stage YOLOv8-b exceeded YOLOv10 by 0.1. Besides, it exceeded TPH-YOLOv5 and YOLO-Fastestv2 by 0.078 and 0.04, respectively. Its accuracy is much higher than those of the Faster R-CNN, YOLOv5, SSD, and CenterNet, as shown in Fig 12. This demonstrated the superiority of the proposed method. The inference time per image of our two-stage YOLOv8-b is 75 ms, which is better than YOLO-Fastestv2 but inferior to all other models. However, it is worth noting the significant improvements in accuracy. Furthermore, for a device with a powerful GPU, the proposed two-stage YOLOv8 can be operated in real-time. The 75-ms inference time had little impact. Therefore, our method achieved a better tradeoff between accuracy and speed, making it more suitable for long-distance ship detection at sea.

**Fig 13:**
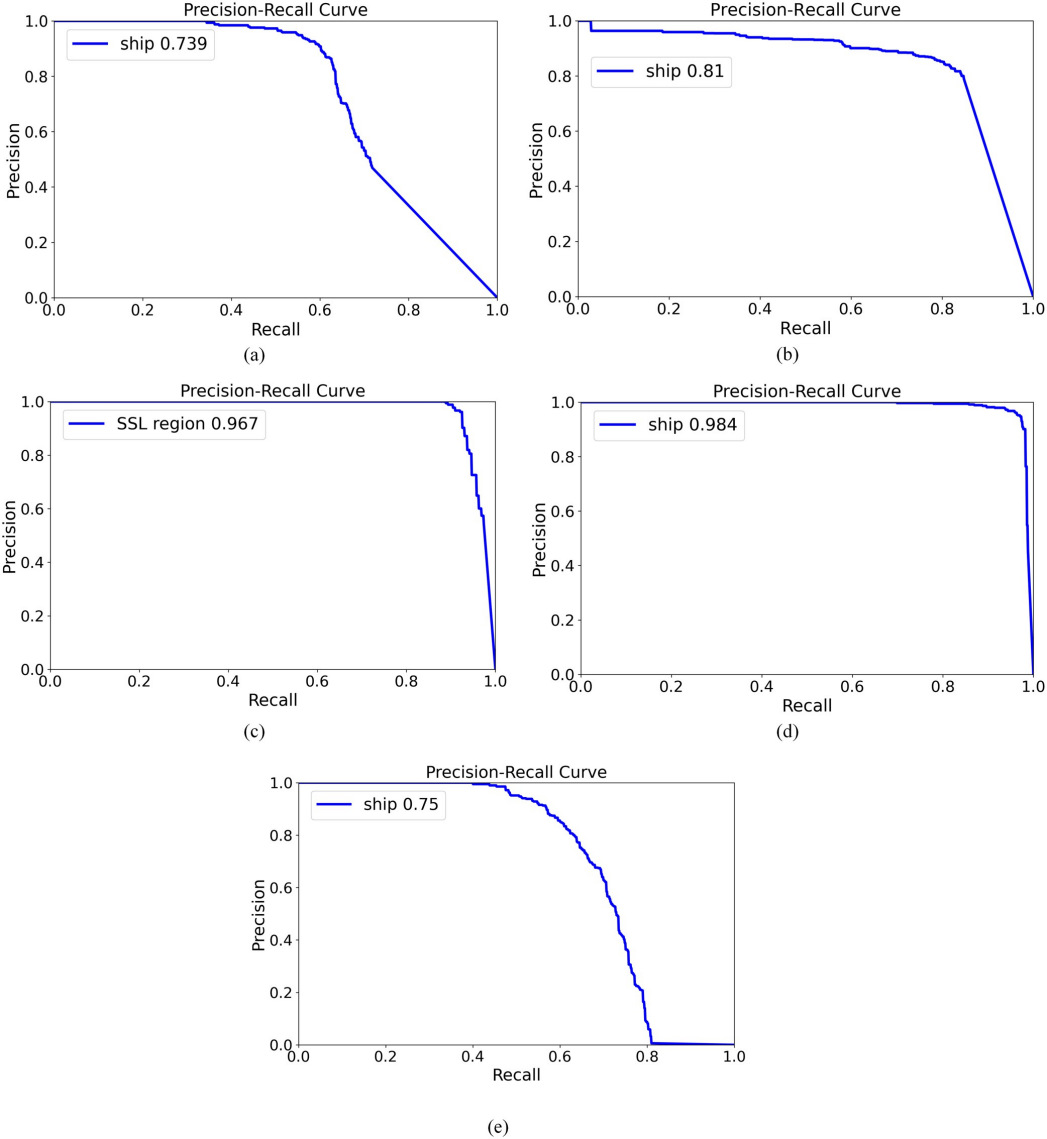
Precision-recall curves of proposed methods, YOLO-Fastestv2, and YOLOv8. **(a)** YOLOv8; **(b)** YOLO-Fastestv2; **(c)** the first stage of our method with an input size of 128; **(d)** the second stage of our method; **(e)** YOLOv10.

Since recall also needs to be considered, and YOLOv8, YOLO-Fastestv2, and YOLOv10 are superior to other compared methods, we compared these three methods with our method simultaneously in terms of precision and recall. The results are shown in Fig 13. In the second stage of our method, the AP_50_ of ships in sliced patches is 0.984. Considering the results after ship relocation in the SSL region, the AP_50_ of ships is 0.879. In the first stage of our method, the AP_50_ of the SSL region is 0.967. Thus, the joint AP_50_ of a ship in the original images is 0.85 (= 0.967×0.879). Our method outperforms YOLOv8, YOLO-Fastestv2, and YOLOv10 in terms of precision-recall curve, demonstrating superior performance.

**Fig 14:**
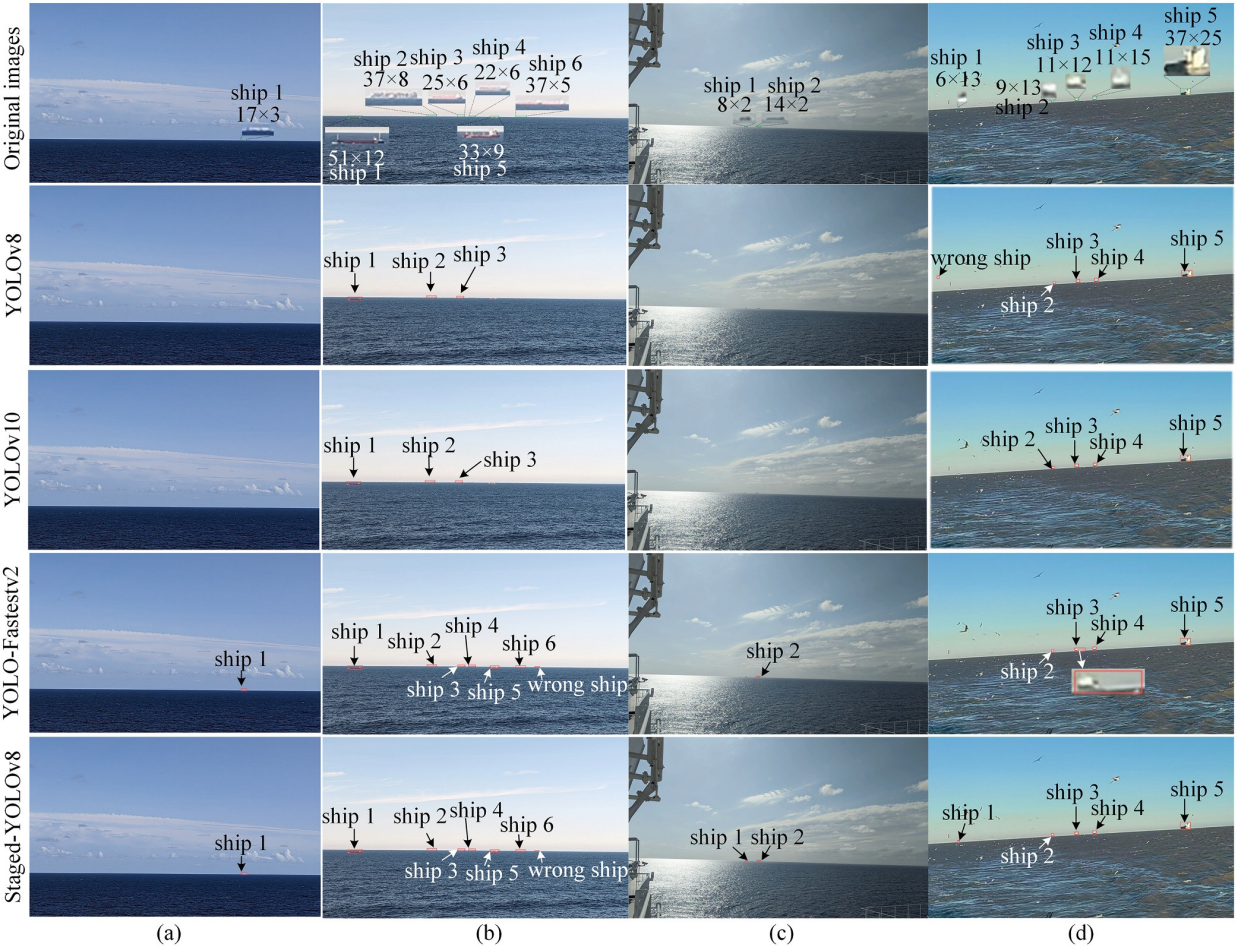
Visualization of the detection results of our methods and YOLOv8. **(a)** there is one ship with a pixel size of 17 × 3; **(b)** there are six ships; **(c)** there are two ships, the smallest of which is only 8 × 2 pixels; **(d)** there are five ships, with many seabirds, are around the SSL.

However, YOLOv8, YOLO-Fastestv2, and YOLOv10 had better accuracy than Faster R-CNN, YOLOv5, SSD, CenterNet, and TPH-YOLOv5; thus, we visualized the detection results of YOLOv8, YOLO-Fastestv2, YOLOv10, and our methods. This visualization is illustrated in Fig 14. It can be seen that our method can rightly detect a long-distance ship with few pixels. As shown in Fig 14a, the ship target has a pixel size of 17 × 3, and YOLOv8 and YOLOv10 cannot detect the ship target, whereas YOLO-Fastestv2 and the proposed method can. In Fig 14b, YOLOv8 and YOLOv10 detected only three larger targets, whereas YOLO-Fastestv2 and our method detected all but one incorrect target. In Fig 14c, because the size of the left target is only 8 × 2 pixels, YOLO-Fastestv2 only detects the larger target, and YOLOv8 and YOLOv10 ignore both targets. Under the influence of seabirds, YOLOv8 misdetects the seabird as a ship and ignores its first target. YOLO-Fastestv2 and YOLOv10 did not detect the first target. However, our method correctly detected all the targets, as shown in Fig 14d. A large portion of seabirds in the sky did not appear in the ship detection of the second stage, owing to the first stage of filtration of the SSL region detection. Therefore, the proposed method can reduce seabird misidentification. In addition, the features of small targets were preserved because we adopted a slicing technique. However, for YOLOv8, YOLOv10, and YOLO-Fastest, many features of the small targets in the original high-resolution images disappeared after the images were resized to 640 × 640 pixels. This demonstrates the superiority of the proposed method in preserving the features of long-distance ships.

## 5. Conclusions

Precise and reliable ship detection methods are crucial for the visual perception of intelligent ships. When a distant ship is located far away from the intelligent vessel, it may appear significantly smaller in the visual image, posing challenges for detection with current object detection models. In this study, we combined the advantages of traditional methods and deep-learning-based methods and proposed a novel method to realize ship detection over long distances. Based on the popular object detection model, YOLOv8, and the slicing technique, we designed a two-stage method. We adopted YOLOv8, which has excellent performance, to detect the SSL region containing potential ship objects in the first stage. The second stage used another YOLOv8 and a slicing technique to detect a ship in the SSL region, which was obtained in the first stage. We constructed a ship dataset with 1871 images captured using a high-definition camera from a distant perspective at sea. Using the two-stage structure, the accuracies of Faster R-CNN, YOLOv5, and YOLOv8 improved significantly, although the detection speed degraded to some extent. For a GPU device with a good performance, the inference time loss is negligible. This demonstrates the effectiveness of the proposed two-stage strategy. In addition, our method outperformed other state-of-the-art models in terms of accuracy and exhibited a good trade-off between accuracy and speed. This makes deep-learning methods reliable when applied to the visual perception of intelligent ships.

**Fig 15:**
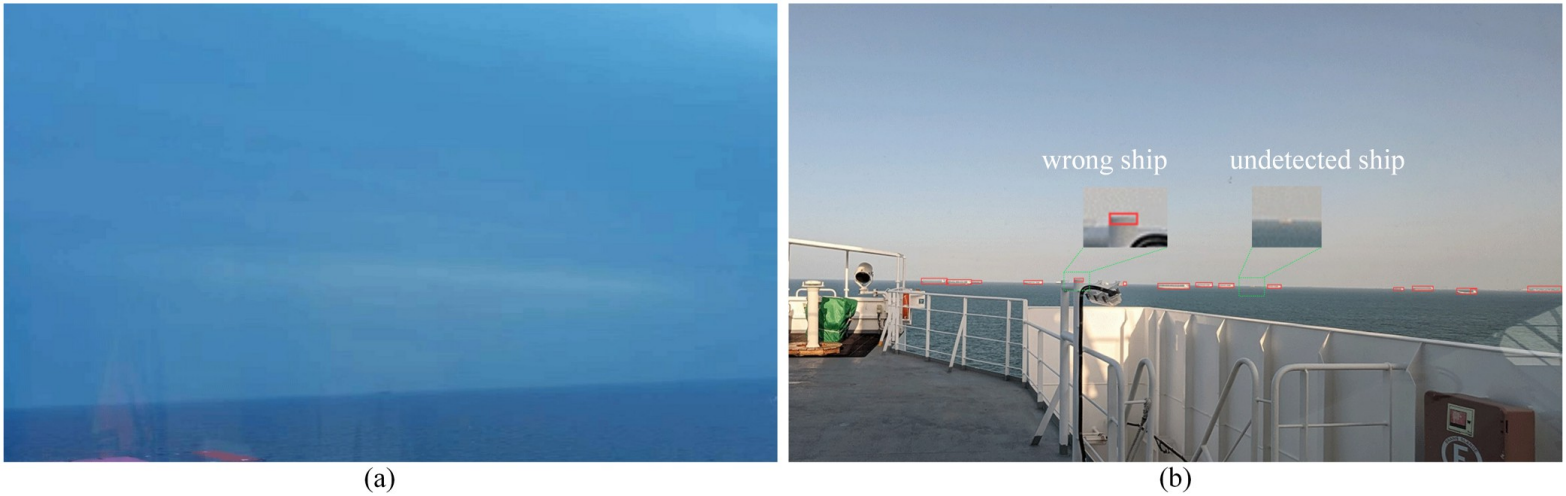
Failure cases of our methods. **(a)** ships are in the rain; **(b)** other objects exist near the SSL and some ships have the same background as the sky.

However, our approach may also fail in several extreme cases, such as rain, objects near the SSL, and extremely small ships with the same background as the sky. As shown in Fig 15a, when the ship to be detected is in the rain, it appears very blurry. In this case, it is not even possible to see with the eyes, let alone with machine vision. In addition, if there are other objects near the SSL, ships may be misdetected. Because these objects have the same features as real ships. When some ships have the same background as the sky, they blend into the background and may not be detected, as shown in Fig 15b. Thus, we should install the camera in a place with a wide field of view to avoid objects on the ship appearing in the view. In addition, try to avoid using automatic ship detection on rainy days. In future studies, we will further improve the detection speed of this method for deployment on embedded devices. At the same time, to solve the above failure cases, we will try to further improve the accuracy of the method by using multiple sensors.
